# Application of Fluorescence in situ Hybridization in Malignant Hemopathies: First Experience from Albania

**DOI:** 10.2478/bjmg-2026-0006

**Published:** 2026-10-14

**Authors:** D Roko, A Babameto-Laku, A Perolla, E Calliku

**Affiliations:** Faculty of Medicine, “Mother Teresa” University Hospital Center, Clinical Genetics Laboratory Service, Tirana; Faculty of Medical Sciences, Albanian University, Tirana; Faculty of Medicine, “Mother Teresa” University Hospital Center, Hematology Service, Tirana

**Keywords:** Fluorescence in situ hybridization, probes, personalized medicine, targeted treatment, prognostic factor

## Abstract

**Introduction:**

Genetic alterations in genome organization are essential for the clinical evaluation of hematological disorders. Fluorescence in situ hybridization (FISH) enables the detection and determination of the frequency of genetic abnormalities.

**Objectives:**

This study aims to apply selected probes as a diagnostic approach to aid in establishing diagnosis, predicting prognosis, and monitoring disease progression.

**Results:**

Fluorescence microscopy analysis of inter-phase cells was undertaken at the Laboratory of Molecular Cytogenetics, within the Clinical Genetics Laboratory Service, in a cohort of 330 adult patients with hematological disorders clinically established by the Hematology Service at QSU “Mother Teresa,” Tirana, Albania.

Detection of the *BCR-ABL1* genetic rearrangement was observed in 50 (40%) patients with CML, providing assistance in establishing a rapid diagnosis and monitoring patient response to the treatment provided. Among AML patients with the PML-RARA genetic rearrangement, 11 (25%) achieved complete remission in a short period following targeted therapy, resulting in a reduced risk of mortality.

The deletion of *TP53* gene identified in a group of 44 (50%) CLL patients represents a major clinical challenge due to its association with a poor prognosis, resistance to the given treatment and an aggressive disease with frequent relapses.

**Conclusions:**

Through the identification of specific genetic mutations, molecular cytogenetics contributes to early diagnosis, improving the prognosis and choosing the most appropriate treatments, significantly improving the survival and quality of patient’s life.

## INTRODUCTION

Malignant hemopathies, commonly known as blood cancers or hematological malignancies, are a diverse group of neoplasms that originate in the blood-forming tissues (bone marrow) or the cells of the immune system (lymphatic system). They occur when genetic mutations cause blood cells to grow uncontrollably, interfering with the production of normal cells [1].

Hematological malignancies have historically been a pioneer among cancers in use of genetic analysis, particularly for diagnosis and classification. Genetic characterization and changes in genome organization are crucial in the clinical evaluation of almost every form of hematological malignancy. Molecular diagnosis is an important diagnostic tool in the diagnosis and their management [2].

The molecular characterization of nonrandom recurrent cytogenetic abnormalities has identified numerous disease-related genes involved in myeloid and lymphoid malignancies. Molecular cytogenetic analysis has become essential for disease diagnosis, classification, prognostic stratification, and treatment guidance [3]. Fluorescence In Situ hybridization (FISH) has greatly enhanced the field and enabled a more precise determination of the presence and frequency of genetic abnormalities.

After overcoming numerous challenges, FISH technology has now been introduced for the first time in the Molecular Cytogenetic Laboratory, Clinical Genetic Laboratory Service, University Hospital Center “Mother Teresa”, Tirana, Albania. FISH analysis is useful in identifying genetic abnormalities undetectable by conventional chromosomal analysis and monitoring residual disease during treatment and follow-up [4].

The aim of this study is to use proper FISH probes such as BCR/ABL1, AML1/ETO, PML/RARA, IGH/MYC, P53 etc as a diagnostic tool to understand the pathophysiology, diagnosis, treatment, prognosis, and monitoring the disease activity in Chronic Myeloid Leukemia (CML), Acute Myeloid Leukemia (AML), Chronic Lymphoblastic Leukemia (CLL), Acute Lymphoblastic Leukemia (ALL), Myelodysplasia (MDS), Multiple myeloma and Lymphoma cases. These probes are presented in table 1.

**Table 1.** Probes used in our service for patients with malignant hemopathy.

**No.**	**Probe**	**Associated hematological malignancies**
1	BCR/ABL1 translocation	CML, ALL
2	AML1/ETO translocation	AML
3	PML/RARA translocation	AML
4	IGH/MYC translocation	ALL, Lymphoma
5	TEL/AML1 translocation	ALL
6	IGH/CCND1 translocation	CLL, Lymphoma
7	MLL (KMT2A) breakapart	ALL, AML
8	E2A breakapart	ALL
9	CDKN2A breakapart	ALL, AML
10	Del (7q) Deletion	AML, MDS
11	Del (20q) Deletion	AML, MDS
12	P53 Deletion	ALL, AML, CLL, Lymphoma, MDS, MM
13	ATM Deletion	CLL
14	Del (5q) Deletion	AML, MDS
15	13q14.3 Deletion	CLL, MM

## MATERIALS AND METHODS

A group of 330 patients with known hematological disorders were studied. Firstly, patients have been consulted and diagnosed clinically in the Hematology Service at the University Hospital Centre “Mother Teresa”, Tirana, Albania. All patients are referred for molecular cytogenetic analysis in the Clinical Genetic Laboratory Service. These records belonged to patients diagnosed with malignant hemopathies between 2021 and 2025. The participants ranged in age from 24 to 81 years, with a male to female ratio of 195:135.

Patients were analyzed using a panel of 15 FISH probes, including D13S319, a molecular marker located on chromosome 13 (13q14), commonly used for the detection of deletions involving this region.

Peripheral blood samples were collected in prewashed syringes with sodium heparin from patients referred for cytogenetic evaluation. All samples were processed within 24 hours of collection. FISH analysis was performed using commercially available dual-fusion translocation, break-apart and deletion probes (Cytocell, Cambridge, UK) according to the manufacturer’s instructions.

Briefly, peripheral blood lymphocytes were cultured using standard molecular cytogenetic protocols. Interphase nuclei were prepared following hypotonic treatment with 0.075 M KCl and fixation in methanol-acetic acid (3:1). Cell suspensions were dropped in clean glass slides and air-dried.

Slides were pretreated in 2×SSC, dehydrated through a graded ethanol series, and air-dried. The probe was applied to the samples, followed by co-denaturation and overnight hybridization at 37°C in a humidified dark chamber. Post-hybridization washes were performed according to the manufacturer’s instructions using SSC buffers of appropriate stringency. Finally, the slides were counterstained with DAPI/antifade solution, cover slipped, and analyzed by fluorescence microscopy, equipped with appropriate filter sets and imaging software (Meta Class). For each probe set, a minimum of 100 interphase nuclei were evaluated.

The sensitivity of the assay was dependent on probe design, hybridization efficiency, and the proportion of abnormal cells within the sample.

For the detection and quantification of transcripts of the *BCR::ABL1* (p210) gene fusion, specifically the *BCR* (b2a2 and b3a2) and *ABL1* genes, in the tested samples, the following procedure was used:

1. RNA extraction using the PureLink Total RNA Kit (Invitrogen, K156001).
2. Quantitative real-time PCR using the BCR-ABL M-bcr Real-Time Quant kit (Sacace Biotechnologies).

The *ABL1* gene was used as the reference gene. For the quantification of accumulated products, quantitative standards QS1 bcr-abl/N-abl, QS2 bcr-abl/N-abl, QS3 bcr-abl/N-abl, QS4 bcr-abl/N-abl, and QS5 bcr-abl/N-abl included in the kit were used. This method detects the *BCR::ABL1* M-bcr fusion, which accounts for approximately 95% of *BCR::ABL1* gene rearrangements in patients with Chronic Myeloid Leukemia.

Although bone marrow aspirate is generally considered the preferred sample for FISH analysis in hematological malignancies, peripheral blood was used in this study due to practical and logistical considerations (limited availability or restricted collection of bone marrow samples and less invasive for patients).

In disorders such as Chronic Myeloid Leukemia, circulating malignant cells are typically present in sufficient numbers to allow reliable detection of cytogenetic abnormalities, including *BCR-ABL1* rearrangements, using peripheral blood samples. Nevertheless, bone marrow remains the gold standard for comprehensive cytogenetic evaluation, and efforts are currently underway to implement bone marrow based FISH analysis in our laboratory in order to further improve diagnostic accuracy and align with international recommendations.

## RESULTS

A total of 330 patients with suspected hematological malignancies were analyzed using FISH technology. Abnormal findings were identified in 127 patients (38.4%). Among the 123 patients diagnosed with Chronic Myeloid Leukemia (CML), 53 were newly diagnosed, while 70 were in the remission phase. The BCR/ABL1 translocation associated with the Philadelphia chromosome was detected in 50 patients (40.6%) using dual-fusion probes specific for the t(9;22) (q34;q11) rearrangement. This translocation was present in 42 of 53 newly diagnosed patients (79.2%) and in 8 of 70 patients in remission (11.4%)

Cytogenetic abnormalities were detected in 49 out of 88 patients (55.6%) diagnosed with Chronic Lymphocytic Leukemia (CLL). The most frequent alteration was deletion of *TP53* (41 cases). Combined deletion of *TP53* and *ATM* was observed in 3 patients, isolated *ATM* deletion in 3 patients, and deletion of D13S319 in 2 patients. PML/RARA rearrangement, consistent with t(15;17) (q24;q21), was identified in 25% (11/44) of AML patients.

In the cohort of 15 ALL patients, 3 cases (20%) demonstrated chromosomal abnormalities: *BCR/ABL1* translocation was observed in two patients, whereas one patient showed an MLL (*KMT2A*) rearrangement detected by break-apart probe analysis. Among 35 patients with Myelodysplastic Syndrome (MDS), 7 (20%) demonstrated cytogenetic abnormalities. Detected alterations included: combined 5q and 7q deletions (1 case), 5q with *TP53* deletion (1 case), isolated 5q deletion (1 case), isolated 7q deletion (1 case), isolated *TP53* deletion (2 cases). *TP53* deletion was identified in 6 of the 17 patients with multiple myeloma, corresponding to a frequency of 35.2%. In the lymphoma cohort, *TP53* deletion was observed in 1 out of 8 patients (12.5%). These results are presented in table 2.

**Table 2.** Results of patients with malignant hemopathy using molecular cytogenetic probes.

**Diagnose**	**Patients**	**Positive results**	**Probes**
Chronic Myeloid Leukemia	123	50 (40%)	BCR/ABL1 translocation (50)
Chronic Lymphocytic Leukemia	88	49 (55,6%)	P53 deletion (41) P53 deletion + ATM deletion (3) ATM deletion (3) D13S319 deletion (2)
Acute Myeloid Leukemia	44	11(25%)	PML/RARa translocation (11)
Acute Lymphocytic Leukemia	15	3(20%)	BCR/ABL1 translocation (2) MLL breakapart (1)
Myelodysplastic Syndrome	35	7(20%)	5q deletion, 7q deletion (1) 5q deletion, P53 deletion (1) 5q deletion (1) 7q deletion (1) P53 deletion (3)
Multiple myeloma	17	6 (35,2%)	P53 deletion (6)
Lymphoma	8	1 (12,5%)	P53 deletion (1)
**TOTAL**	330	127 (38,4%)	

**Figure 1.**
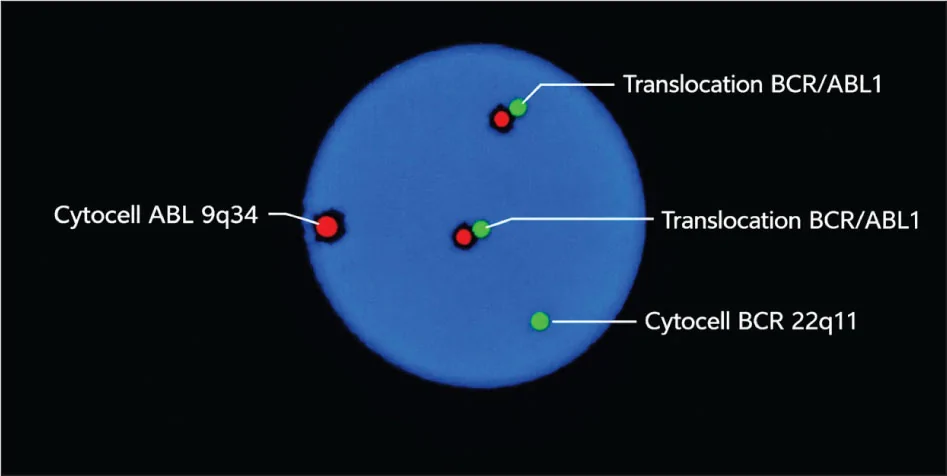
*BCR-ABL/ABL1* translocation, dual fusion probe

**Figure 2.**
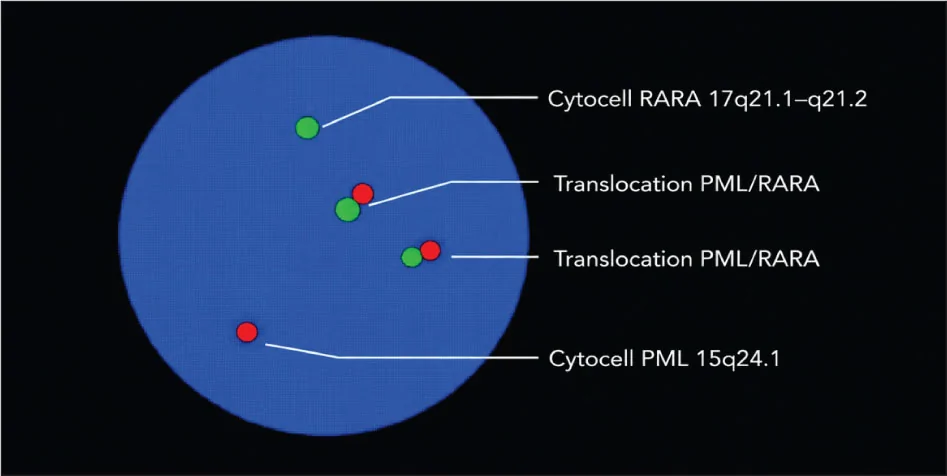
PML/RARa translocation, dual fusion probe

## DISCUSSION

The findings confirm clinical utility for targeted FISH probes in the routine diagnostic workup of malignant hemopathies particularly when rapid detection of recurrent genomic abnormalities is required.

A limitation of this study is that FISH analysis was performed on peripheral blood samples rather than on bone marrow aspirates, which are considered the gold-standard specimen for the detection of cytogenetic abnormalities in certain hematologic malignancies. Although peripheral blood sampling offers a less invasive and more practical approach, it may have lower sensitivity for detecting low-level clonal populations, particularly in disorders such as Multiple Myeloma and Myelodysplastic Syndrome (MDS).

In patients with Chronic Myeloid Leukemia (CML), the detection rate of *BCR-ABL1* rearrangement was 40%. Presence of this fusion, resulting from t (9;22) (q34; q11), is the molecular hallmark of CML and represents a critical diagnostic and therapeutic target for monitoring disease response to treatment. Identification of this rearrangement supports the essential role of FISH in confirming the diagnosis, especially in cases where conventional cytogenetics may be inconclusive or metaphases are insufficient.

In our laboratory, Real-Time PCR (qPCR) analysis was performed in patients with CML who were in remission and had tested negative for *BCR-ABL1* rearrangement by FISH. Real-Time PCR, due to its high sensitivity, allows for the identification of minimal residual disease (MRD), providing a more accurate assessment of molecular response. The implementation of this method is essential for precise disease monitoring, evaluation of treatment efficacy, and early detection of potential relapse in CML patients.

Findings similar to our results have been reported in other published studies, where the detection of *BCR-ABL1* translocation by FISH presents variability depending on the population studied, the methodology used, and the patient inclusion criteria [5,6].

Chronic Lymphocytic Leukemia (CLL) demonstrated the highest proportion of positive results (55.6%) with *TP53* deletion being the most frequent abnormality. Deletions involving 17p13 (*TP53* locus) are well-established adverse prognostic markers and are associated with resistance to conventional chemotherapy that remain a major challenge. The disease is more aggressive and progresses faster [7,8]. The detection of isolated *ATM* deletions and combined *TP53/ATM* deletions further highlights the genetic heterogeneity of CLL and underlines the importance of comprehensive FISH probes in risk stratification [9].

Although clinical outcome data were not available in our study, the coexistence of *TP53* and *ATM* deletions has been recognized as a high-risk genetic feature in hematologic malignancies.

In addition to the double *TP53/ATM* deletion, numerical abnormalities such as monosomy 11 and trisomy 14 were also identified in one patient. Careful monitoring by modifying the dose of maintenance medication according to certain protocols and personalized therapeutic approaches should be essential for patients with complex cytogenetics [10].

In Acute Myeloid Leukemia (AML), 25% of cases harbored the PML-RARA rearrangement, corresponding to acute promyelocytic leukemia (APL). This finding is of particular clinical relevance, as rapid identification of PML-RARA is critical due to the distinct therapeutic approach. The results emphasize the value of FISH as a rapid diagnostic tool in acute leukemia settings, where timely molecular confirmation can directly impact patient outcomes [11]. The target therapy received has significantly improved the prognosis for APL patients with high rates of remission and overall survival. Three patients achieved complete remission after targeted therapy, indicating a favorable therapeutic response. In one patient numerical abnormalities such as trisomy 8, trisomy 21, tetrasomy 21 were also identified [12].

Among Acute Lymphoblastic Leukemia (ALL) cases, abnormalities were detected in 20% of patients, including *BCR-ABL1* rearrangements and one *KMT2A* (MLL) rearrangement identified by break-apart probe. Both alterations are associated with high-risk disease and poorer prognosis. Their identification is essential for risk-adapted therapy, reinforcing the importance of incorporating FISH analysis into the diagnostic algorithm of ALL [13].

In Myelodysplastic Syndrome (MDS), 20% of patients showed cytogenetic abnormalities, including deletions of 5q, 7q, and TP53 deletion [14]. *TP53* deletion was the most common abnormality, identified in 3 of 7 cytogenetically abnormal cases of Myelodysplastic Syndrome. These findings may affect diagnostic processes and patient clinical management, advancing precision medicine beyond conventional disease stratification on the basis of clinical features and gene mutations [15]. Isolated del(5q) is associated with a distinct clinical entity and specific therapeutic implications [16], isolated 7q deletion appears to be high risk cytogenetic abnormality associated with progression or relapse of disease [17], while monosomy 7 or del(7q) and TP53 deletion are generally linked to adverse prognosis and higher risk of progression to AML [18]. The detection of combined abnormalities further reflects clonal evolution and genomic instability characteristic of MDS.

In Multiple Myeloma, *TP53* deletion was detected in 35.2% of patients in our cohort. This alteration has been consistently associated in the literature with high-risk disease features and inferior overall survival. In two patients, *TP53* deletion was identified at relapse, a finding that has also been linked to poor clinical outcomes in previous studies. Although the sample size was limited, the observed frequency highlights the potential prognostic relevance of 17p (*TP53*) deletion in plasma cell neoplasms [19, 20].

*TP53* deletion was detected within the lymphoma cohort. Although the number of affected cases was limited, TP53 abnormalities have been associated with aggressive disease behavior in several lymphoma subtypes [21].

## CONCLUSION

Provide a powerful tool to improve and to increase the quality of health care and brings opportunities for further progress in scientific medical research. FISH analysis for the first time in Albania provides a reliable and clinically informative method for detecting cytogenetic abnormalities in hematological malignancies. The use of molecular cytogenetic analysis has become an essential part of the clinical management for diagnostic, classification, prognostic and therapeutic purposes with a very high level of accuracy improving quality of life of patients. Among these examinations, minimal residual disease and target therapy were determined, achieving very good results in the treatment or their complete remission by modifying the dose of maintenance medication according to certain protocols. Integration of FISH with complementary molecular techniques may further enhance diagnostic precision and prognostic stratification for patients in Albania.
